# What Are the Most Important Factors Influencing Springtail *Tetrodontophora bielanensis*?

**DOI:** 10.3390/insects12100858

**Published:** 2021-09-23

**Authors:** Strahinja Mladenović, Jan Materna, Tereza Brestovanská, Jakub Horák

**Affiliations:** 1 Faculty of Forestry and Wood Sciences, Czech University of Life Sciences Prague, Kamýcká 129, 165 00 Praha-Suchdol, Czech Republic; st.mladenovic@gmail.com (S.M.); sllunicko@seznam.cz (T.B.); 2 Krkonoše Mountains National Park, Dobrovského 3, 543 01 Vrchlabí, Czech Republic; jmaterna@krnap.cz

**Keywords:** Norway spruce, European beech, spatial autocorrelation, dead wood, saproxylic microhabitats

## Abstract

**Simple Summary:**

Springtails (Collembola) are organisms which mainly inhabit soil and litter layers but may account for around 25% of the canopy fauna. They can be also found in dead wood, which provides nutrients for these detritivores. The springtail, *Tetrodontophora bielanensis*, dwells in the litter and upper soil layers. In our research, we were focused on the environmental factors influencing this springtail in forests at the landscape and site level. We found that the springtail was positively influenced by the presence of Norway spruce (*Picea abies*) and with greater abundance in the south-eastern part of Krkonoše (Czech Republic). The negative influence of bark coverage, the presence of fungi, and the positive influence of an increasing circumference of tree was observed at the site level.

**Abstract:**

The springtail, *Tetrodontophora bielanensis*, dwells in the litter and upper soil layers. This arthropod mainly inhabits humid litter and soil and prefers a cold climate. We determined the main factors influencing this springtail in forests at the landscape level in Krkonoše and site level in Orlické hory in the Czech Republic. We used passive trunk-tree traps. These traps are highly effective for sampling flightless fauna. We used 128 traps in Krkonoše and 17 traps in Orlické hory. The springtail was significantly positively influenced by the presence of Norway spruce (*Picea abies*) at the landscape level. Springtails’ abundance was, furthermore, influenced by the spatial distribution of the sampling sites. The negative influence of bark coverage and the presence of fungi, and positive influence of an increasing dimension of trees were significant at the site level. We argue for a more diversified management of mountainous forests with respect to forest history. This appears to be also important for mountainous forests in protected areas.

## 1. Introduction

Springtails (Collembola) are considered to be organisms mainly inhabiting soil and litter layers [1]. Nevertheless, forest collembolans might account for around 25% of the canopy fauna [2]. Springtails are also found in dead wood, which provides nutrients for these detritivores [3].

The springtail, *Tetrodontophora bielanensis* (Waga, 1842), inhabits litter and upper soil layers and mainly lives in forests [4]. It is well-known for its chemical defenses and usually climbs on tree trunks in search for food, but only when there is enough moisture [5]. It prefers low temperatures, ideally between 8–11 °C [6], and therefore mostly inhabits mountain ranges. In the lowlands, it lives in close proximity to watercourses, where it can find suitable living conditions [7]. Soil humidity mainly affects the distribution of collembolans at smaller scales [8]. In Europe, the range of *Tetrodontophora bielanensis* extends from the southern Carpathians in Romania, through Ukraine, up to the northern mountain ranges in Poland and the Czech Republic [9]. Similar to other collembolans, this species also plays an important role in decomposition and nutrient cycling [1,10,11] in habitats with dead wood.

Dead wood represents an important habitat for a number of forest organisms. The decomposition process is important for understanding the influence of the forest environment to arthropod communities. Collembola have, in general, a low dispersal ability and can therefore be used to examine habitat suitability [12]. Springtails are mainly subjects of studies dealing with pitfall traps or litter and soil sample extraction [1]. Recent studies have indicated that tree-traps can be successfully used for the evaluation of forest biodiversity, including flightless species [13].

Studies dealing with marginal, or rather neglected, and, furthermore, facultative saproxylic organisms are still scarce [14], even if these organisms could play important roles within this highly studied guild [15]. Springtails are one of those organisms most omitted [1,4,6]. The most important information derived from this study shows the interconnection of two topics. The first is that the springtail is a conspicuous member of a inadequately studied saproxylic taxa. The second is the importance of combining two approaches to the influences of the environment on this taxon, i.e., landscape and site level parameters. We predicted that the studied species distribution (as reflected by the trapping success of its individuals) would be influenced mainly by the tree level characteristics (e.g., species or diameter) at the landscape and site level.

### Main Aims

We investigated the forest environmental factors influencing *Tetrodontophora bielanensis* at two spatial scales. In this study we aimed at answering the following two questions:

1. How do tree characteristics and deadwood presence influence the species at the landscape scale?
2. How is the distribution of the species influenced by forest tree factors at the site level?

## 2. Materials and Methods

### 2.1. Trapping Method

**Figure 1:**
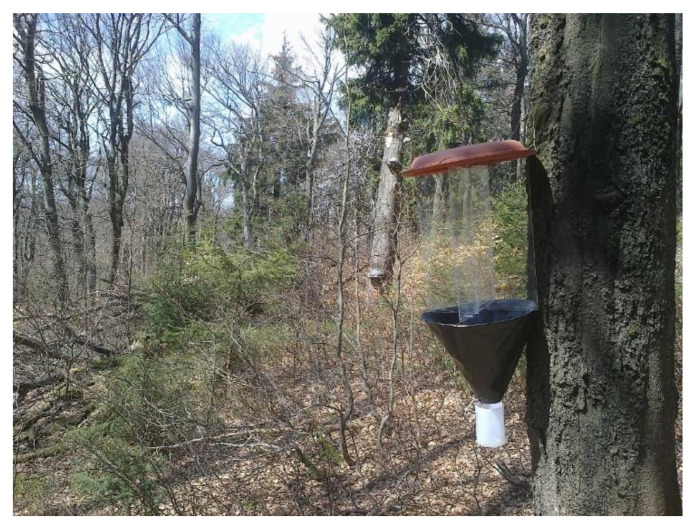
Trunk-tree window trap used in Krkonoše and Orlické hory for trapping of the *Tetrodontophora bielanensis*. Author: Jakub Horák.

We used passive (i.e., non-attractive) window traps placed on tree trunks (Figure 1), which are highly effective for the sampling of even flightless fauna [13]. Each trap consisted of crossed transparent plastic panes (400 × 500 mm), a protective top cover, and a funnel leading down into a container with a solution of water, salt, and detergent. Each trap was placed at a height of 1.3 m, facing south.

### 2.2. Study Areas

#### 2.2.1. Krkonoše—Landscape Level

**Figure 2:**
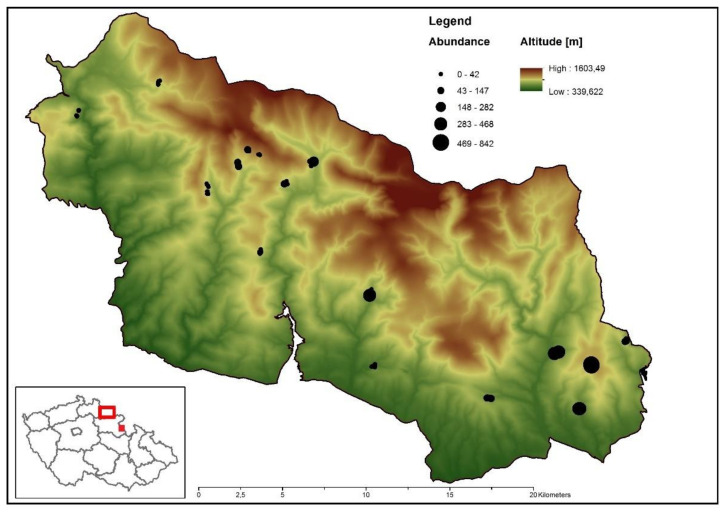
Map showing the study sites. The frame illustrates Krkonoše (large red rectangle) and Orlické hory (small red square). The large area indicates the spatial distribution of the study sites and the trapping success of the *Tetrodontophora bielanensis* (black points) in Krkonoše. Author: Jiří Trombik and Jakub Horák.

The Czech part of Krkonoše covers an area of nearly 454 km^2^. It is located mainly in the area of the National Park Krkonoše, which is the oldest national park in the Czech Republic (founded in 1963). From a historical perspective, the mountains of Krkonoše were mainly covered by European beech (*Fagus sylvatica*) stands. However, in the last centuries, most of the original tree cover has been replaced by the commercially more attractive Norway spruce (*Picea abies*), which is native to the area, but was formerly only present at high altitude. We used 128 traps that were placed throughout the area of Krkonoše. European beech and Norway spruce were equally represented (Figure 2). One half (64) of traps was used in 2015 in the eastern part and the second half in 2016 in the western part. The minimum distance between traps was 50 m.

#### 2.2.2. Orlické Hory—Site Level

We used 17 traps in Orlické Mts (Orlické hory) in 2009. Traps were placed in the core area of Bukačka (Figure 2), an old mountainous spruce–beech forest covering more than 50 ha, at an altitude of approximately in 1000 m above sea level. The area of Bukačka has recently been unmanaged, except for several salvage cuttings over the past decades due to bark beetle outbreaks. European beech and Norway spruce are nearly equally represented; a smaller part this forest consists of grassland [16].

### 2.3. Environmental Variables

#### 2.3.1. Krkonoše

We investigated seven environmental variables. To obtain standardized data at the landscape level, we only sampled living trees without microhabitats.

We studied the differences in the selection between two tree species. In particular, we used a paired design—i.e., traps on spruce (*n* = 64) and beech (*n* = 64) in each studied forest. The circumference of a particular tree was measured at the breast height in cm. Due to the diverse topography of mountainous areas, we determined the influence of topography reflected by altitude in m a.s.l. To assess the effect of canopy openness, each trap was also photographed from the top, using a Canon EF-8–15 mm f/4 L FishEye USM under full foliage. Photographs were evaluated using Gap Light Analyser 2.0 in %. We estimated the amount of dead wood in m^3^ in the surrounding of a sampled tree—i.e., within a 5 m radius (Table 1). As we studied *Tetrodontophora bielanensis* in Krkonoše during two consequent years, we used the study year as a control for potential temporal bias. Autocovariate was used as a control for the potential influence of spatial autocorrelation (see Section 2.4).

#### 2.3.2. Orlické Hory

We evaluated five tree-level predictors that reflect forest tree microhabitats. Namely, we discriminated the tree species between spruce (*n* = 8) and beech (*n* = 9). The stage of decay of the trunk was the second tree-level predictor, with traps on dying trees (*n* = 5), dead trees (*n* = 5), and snags (*n* = 7). We also checked if the trunk was significantly inhabited by wood-inhabiting fungi (i.e., the presence of fruiting bodies; *n* = 13). Subsequently, we estimated the percentage of bark coverage in % on each tree and measured the circumference of the tree in cm at 1.3 m above the ground (Table 1).

### 2.4. Statistical Analyses

Data on the abundance of *Tetrodontophora bielanensis* in Krkonoše were cubic-root transformed to obtain gamma distribution, and data from Orlické were square-root transformed to reach Gaussian distribution. Both dependent variables were first tested for spatial autocorrelation using Geary’s *C* test. The abundance of the springtail was spatially dependent in Krkonoše (*C* = 0.48; *p* < 0.001), but not in Orlické hory (*C* = 0.68; *p* = 0.09).

To control the potential spatial bias, we implemented the autocovariate of the dependent variable in the dataset from Krkonoše, as mentioned above. Temporal bias was controlled using the sampling year in the case of the Krkonoše dataset. To analyze the influence of environmental predictors on the springtail in Krkonoše, we used the generalized linear mixed-effect model (GLMM), with pair of traps (beech and spruce) as a random factor, applying the package MASS. The influence of tree indicators on the springtail in Orlické hory was assessed using the generalized linear model (GLM). Furthermore, we performed predictor selection using the packages nlme, pgirmes, and MASS. The Δ AICc < 2 was used as the criterion for the final GLM selection. The explained variance of the finally selected tree predictors was analyzed by hierarchical partitioning, using the package hier.part. All analyses were performed in R 3.0.2.

## 3. Results

In total, we trapped 18,252 individuals of *Tetrodontophora bielanensis* (7592 in Krkonoše and 10,660 in Orlické hory).

### 3.1. Landscape Scale—Krkonoše

Springtail abundance was higher on spruce than on beech. Species abundance was, furthermore, influenced by the spatial distribution of the study sites. There was strong spatial autocorrelation in the data with springtail abundances being higher in the southeast locations (Figure 2 and Table 2). 

### 3.2. Site Level—Orlické Hory

Site level results indicate that *Tetrodontophora bielanensis* was significantly negatively affected by the presence of fruiting bodies of wood-inhabiting fungi and by increased bark coverage (Table 3).

**Figure 3:**
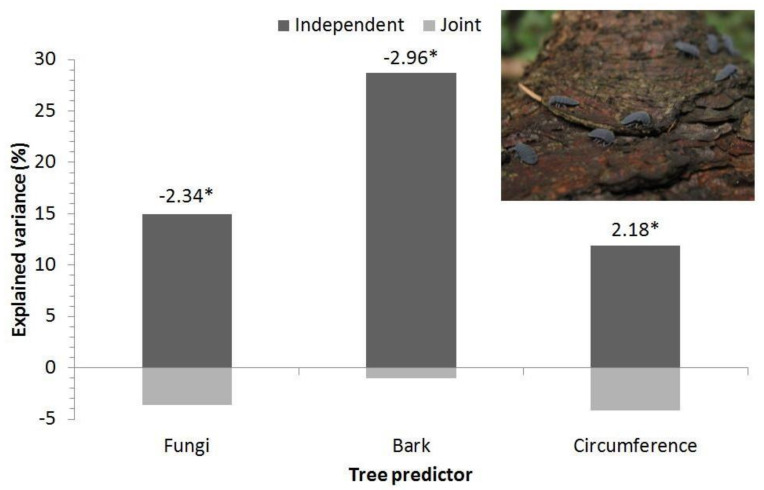
Influences of environmental variables on *Tetrodontophora bielanensis* in Orlické hory after generalized linear model (GLM) model selection. Captions are t-values; * indicates *p* < 0.05 of GLM. Individuals of the springtail on the bark of spruce are shown in the frame.

The negative influences of bark coverage, the presence of fungi, and the positive influence of increasing circumference of tree had significant effects on the abundance of the springtail in Orlické hory after the best subset GLM selection (Figure 3).

## 4. Discussion

In terms of *Tetrodontophora bielanensis* abundance, the effect of a spatial gradient from the northwest to the southeast was the most important factor at the landscape level, followed by the effect of the tree species. The effects of wood-inhabiting fungi, bark coverage, and stem circumference were the highest at the site level.

We investigated the factors measured at the macro- (landscape) and meso- (site) scales affecting this saproxylic springtail. In the case of Krkonoše (landscape level), we eliminated the effect of the tree habitats by using only healthy trees [17], while in the site study, we focused on tree habitats in detail. It is, thus, not surprising that the physical characteristics of trees, representing microhabitats, were more important than tree species or distance between sites [18].

### 4.1. Effects at the Landscape Scale

The spatial aggregation effect on springtail abundance is not surprising at the landscape level—this spatial distribution of species individuals is common in nature [19]. In the wild, animals often live in herds, flocks, or swarms. They are often gregarious and aggregate behaviorally and *Tetrodontophora bielanensis* is not exception. This species is mentioned as gregarious in the literature [20,21], and the landscape suitability in the southeastern part of Krkonoše appears to be subjected to fewer human activities than the northwestern part [22].

*Tetrodontophora bielanensis* also preferred spruce trees. One of the main reasons could be the rough bark of the spruce tree, facilitating the crawling of this flightless arthropod [13]. Nevertheless, this is in contrast with the findings at the site scale, as the species preferred stems without bark. This leads us to infer that the other factors might play a role (such as bark pH or food availability). Alternatively, the aboveground activity of the species increases with increasing humidity [6], and rain makes beech stems unsuitable for crawling, while the bark of spruce trees still facilitates crawling or hiding.

### 4.2. Meso-Scale Overview

The result on the site level precisely reflects the present situation of many forests in Europe. The trees in this part of the study are typical of those of forest stands after bark beetle attack, including large dead and dying trees with variable amounts of remaining bark. Our results show some similarities to studies done on the same site with saproxylic beetles, showing preferences for large trees in both cases. However, contrary to the springtails in our study, beetles showed strong positive relationships with dead wood presence and with fungi. Nevertheless, previous results for some threatened species in the same site indicate similar patterns, mainly driven by the reaction to the circumference of the stem. One threatened click beetle species, *Ampedus auripes*, prefers dead spruce stems without the presence of wood-decaying fungi. Similarly, *Hapalaraea pygmaea* in Orlické hory prefers snags with a large diameter [18].

However, beetles frequently act as vectors for fungi, while fungi serve as food for the beetles, which is also true for other invertebrates [23,24]. When focusing on the preferences of our species, there is evidence that *Tetrodontophora bielanensis* feeds on a large spectrum of fungi (not only wood-inhabiting) [25]. Furthermore, a recent study has revealed the capability of springtails to transport spores on their body surface and in their intestines [26], which poses the question of whether wood-inhabiting fungi are good indicators of the food availability for springtails, as collembolans are often considered to be fungivores in soil food webs [27].

### 4.3. Springtails and Dead Wood Habitats

Besides *Tetrodontophora bielanensis*, the majority of Collembola species are decomposers [28]. Several taxa are considered fully saproxylic [29,30,31]. Saproxylicity is a part of keystone processes of nutrient cycling and decomposing, which means mechanically degrading and feeding on dead wood organic matter [32,33]. Relatively high difference in the number of individuals between two study areas could be explained by the state of decomposition of the individual tree in the case of meso-scale study. In the case of large-scale study, this effect was eliminated. However, there is still a lack of knowledge on springtails regarding particular habitats in forests, such as dead wood [34,35]. Thus, it is not easy to compare our results with other studies on collembolans, mainly because most studies on springtails focused on differences between forest sites or compared forests with open habitats [4]. Trunk tree traps appear to be a highly effective method for the collection of similar species of arthropods (e.g., other springtails, mites, or ants), which has previously been indicated on flightless beetles [13].

## 5. Conclusions

*Tetrodontophora bielanensis* was significantly and positively influenced by the Norway spruce over the European beech. This is important information as the Norway spruce is one of the most abundant tree species in European forests [36]. Spatial distribution of the springtail might also reflect the potential impacts of the forest management history [13,37,38]. This is also highlighted by the preference for saproxylic tree habitats. We do not know much about the status of populations of this springtail species in the study area, but its requirements reflect the need for a mosaic of disparate tree habitats in forests and for facultative saproxylic species. Thus, our results argue for a more diversified management of mountainous forests—mainly, the retention of dead and dying trees as habitat for saproxylic organisms like *T. bielanensis*.

## Figures and Tables

**Table 1:** Values of continuous environmental variables measured in Krkonoše and Orlické hory.

Level	Variable	Mean	Standard Error
Landscape	Altitude (m a.s.l.)	800.8	12.32
	Circumference (cm)	154.92	3.46
	Dead wood (m^3^)	2.17	0.31
	Canopy openness (%)	16.11	0.53
Site	Bark coverage (%)	74.71	5.89
	Circumference (cm)	161.59	10.14

**Table 2:** Influences of environmental predictors on *Tetrodontophora bielanensis* abundance in Krkonoše, using the generalized linear mixed effects model (significant *p*-values are in bold).

Environmental Variable	*t*	*p*
Altitude	1.18	0.24
Tree species (spruce)	2.61	**0.011**
Tree circumference	−1.73	0.09
Dead wood	−0.99	0.33
Canopy openness	0.23	0.82
Year	1.23	0.22
Autocovariate	−4.24	**<0.001**

**Table 3:** Influences of tree indicators on *Tetrodontophora bielanensis* abundance in Orlické hory, using the generalized linear model (significant *p*-values are in bold).

Environmental Variable	*t*	*p*
Tree species (spruce)	−0.92	0.38
Tree decay stage	0.28	0.78
Fungi	−2.26	**0.045**
Bark	−2.54	**0.028**
Tree circumference	1.82	0.10

## Data Availability

Data are available on request.
